# Wafer-Scale Deposition of 2D SnSe with Monolayer-Thickness Precision

**DOI:** 10.1021/acs.nanolett.6c01711

**Published:** 2026-08-20

**Authors:** Lauren M. Garten, Hannah E. Wolfe, Jeremy Knight, Jonathan R. Chin, Joshua D. Wahl, Marshall B. Frye

**Affiliations:** School of Materials Science and Engineering, 1372 Georgia Institute of Technology, Atlanta, Georgia 30332-0245, United States

**Keywords:** 2D Materials, Wafer scale, SnSe, Pulsed laser deposition, Mirror raster

## Abstract

2D SnSe presents considerable opportunities for transistors, optoelectronics, and photonics if wafer-scale deposition with monolayer thickness control can be achieved. This work answers this critical need by developing a mirror rastering pulsed laser deposition (PLD) approach that enables wafer scale coverage of SnSe with monolayer thickness precision and atomic scale surface roughness (0.29 nm). SnSe thin films are deposited onto 7.5 × 5 cm flexible mica substrate in monolayer increments from 3.5 to 6.1 nm by mirror rastered PLD. All films exhibit stoichiometric (200) oriented *Pnma* SnSe with consistent phase and thickness across the length of the wafer. Furthermore, sheet resistance is constant with an average of 0.52 GΩ/sq across a 6 cm span and remains stable upon mechanical flexing. This work demonstrates the wafer-scale SnSe deposition with precise thickness control needed to enable next-generation 2D electronics and optoelectronics.

## Graphical abstract

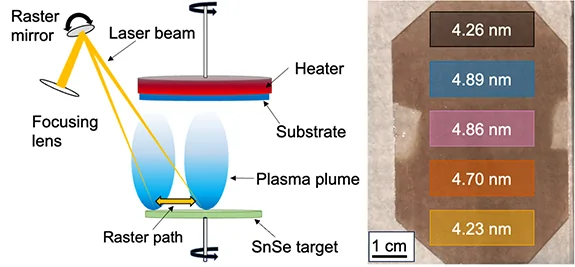

The 2D monochalcogenides hold significant promise for nanoscale electronics once wafer-scale deposition with thickness control can be achieved.^1^ Mechanical exfoliation, which has long been used to isolate 2D materials,^2^ produces flakes with limited lateral coverage and variable thicknesses in monochalcogenides.^3^ Direct film deposition methods that offer layer control with lateral coverage at the wafer scale are critically needed for the integration of these highly anisotropic 2D materials into field-effect transistors, optoelectronics, and photonics.^4,5^

Wafer-scale deposition of 2D materials has been demonstrated in transition metal dichalcogenides, such as MoS_2_ and WS_2_, by metal organic chemical vapor deposition (MOCVD) and pulsed laser deposition (PLD),^6,7^ enabling efficient photodetection and transistors.^8^ The question remains whether the methods that exist for transition metal dichalcogenides can be translated to highly anisotropic monochalcogenides, which exhibit stronger interlayer binding energies and have emergent properties with thickness scaling: including bandgap tunability, second harmonic generation, piezoelectricity, and ferroelectricity.^4,9–13^

As a 2D monochalcogenide that forms in the highly anisotropic *Pnma* crystal structure, SnSe provides an ideal model system to develop wafer-scale deposition.^14,15^ SnSe can be processed at low enough temperatures (475 °C) to facilitate integration with silicon microelectronics.^16^ Previous solution-processed SnSe films rely on multistep reactions to decompose Sn_3_Se_4_ to SnSe but result in SnSe_2_ secondary phases and use hepatotoxic dimethylformamide (DMF),^15,17^ prompting the search for safer, more readily scalable methods.

There are also reports of SnSe thin-film deposition by chemical vapor deposition (CVD),^18^ molecular beam epitaxy (MBE),^19^ and pulsed laser deposition (PLD),^20^ but no method yet offers the combination of phase purity, crystallographic orientation, and thickness control at the wafer scale. For example, MBE offers atomic layer control,^9,19^ but SnSe films thinner than 6 monolayers become discontinuous islands.^19^ Similarly, during deposition by CVD, SnSe tends to form discrete micrometer-sized islands for films below 10 nm.^21,22^ PLD has enabled high-quality SnSe films thicker than 50 nm^23,24^ and the deposition of isostructural 2D chalcogenides, such as GaSe and SnS,^25^ with precise layer control.^26^ However, scaling the lateral scale of monochalcogenides to the wafer scale while enabling monolayer scale precision is a key limitation of translating 2D monochalcogenides to technological relevance.

This work describes the development of a mirror-raster-pulsed laser deposition process that enables wafer-scale coverage of SnSe with monolayer control. Using mirror rastering, SnSe films are deposited across a flexible 7.5 × 5 cm wafer with consistent crystal structure and submonolayer thickness variations. Thus, this work provides the framework to enable next-generation 2D electronic and optoelectronic devices such as complementary metal oxide semiconductor devices and ferroelectric field effect transistors.

Since previous stabilization of SnSe by pulsed laser deposition (PLD) focused on films thicker than 50 nm,^23,24^ we need to establish the growth window for *Pnma* SnSe with a (200) orientation for films thinner than 10 nm. X-ray diffraction (XRD) was measured as a function of substrate temperature for target film thicknesses of 6 nm, as shown in Figure S1a. While all films show oriented SnSe with the desired phase as the temperature increased from 475 to 500 °C, the growth mode transitions from layered to islanded morphology, as shown in Figure S1b,c. Furthermore, the AFM data demonstrate a root-mean-square roughness (*R*_q_) of 1.41 nm for films deposited at 500 °C and an *R*_q_ of 0.64 nm for films deposited at 475 °C. The change in morphology is attributed to adatom desorption at elevated temperatures. Next, the impact of laser fluence was tested, ranging from 1.4 to 2.1 J/cm^2^. XRD of these films is shown in Figure S2a, where a deposition fluence of 1.6 J/cm^2^ was identified as optimal for film crystallinity. Films deposited with these conditions are highly oriented, shown by the narrow rocking curve full-width at half-maximum (fwhm) of 0.12° in Figure S2b. The alignment between SnSe and mica was measured using in-plane XRD, as shown in Figure S3. The in-plane XRD reveals 6 distinct in-plane growth orientations that are aligned with the underlying substrate, which is expected due to the pseudohexagonal symmetry of mica.^27^ Similar orientation relationships between substrates and SnSe have previously been reported for other growth methods.^9,28,29^

To expand the lateral range of SnSe films across a wafer scale, we developed a mirror rastering PLD procedure. In this approach, the laser beam is swept along the target by rastering a mirror, moving the point of maximum atomic flux across the substrate. When combined with substrate rotation, this technique is expected to reduce local thickness variations and improve deposition uniformity across larger areas.^30^ Figure 1a provides a schematic of how the rastering of the laser mirror enables broad wafer coverage by shifting the position of the ablated plume across the substrate. Using this mirror rastering approach, SnSe films were deposited onto mica substrates using the conditions found to stabilize the phase with layered growth. Figure 1b shows an image of the resulting 4.5 nm thick SnSe thin films deposited across a 7.5 × 5 cm substrate area. The clear regions at the top and bottom of the film were masked with the substrate holder. Color gradients were not observed across the deposition area, indicating uniform coverage of the films. Since SnSe is predicted to exhibit and has experimentally realized thickness-dependent properties, it is critical to have precise control of the film thickness. The film thickness was controlled by changing the number of pulses from 275 (targeted thickness = 6 monolayers) to 3000 (targeted thickness = 55 monolayers). The film thicknesses were then verified by X-ray reflectivity (XRR) measurements. The XRR data and corresponding fits in Figure 1c show the layer control provided by the mirror rastering PLD approach. The thickness is modulated from 3.5 to 6.1 nm in monolayer increments (0.575 nm).^9^ The level of thickness control achieved here is critically needed to probe emergent properties with reduced thickness, such as piezoelectricity, ferroelectricity, and second-harmonic generation.^31,32^

**Figure 1:**
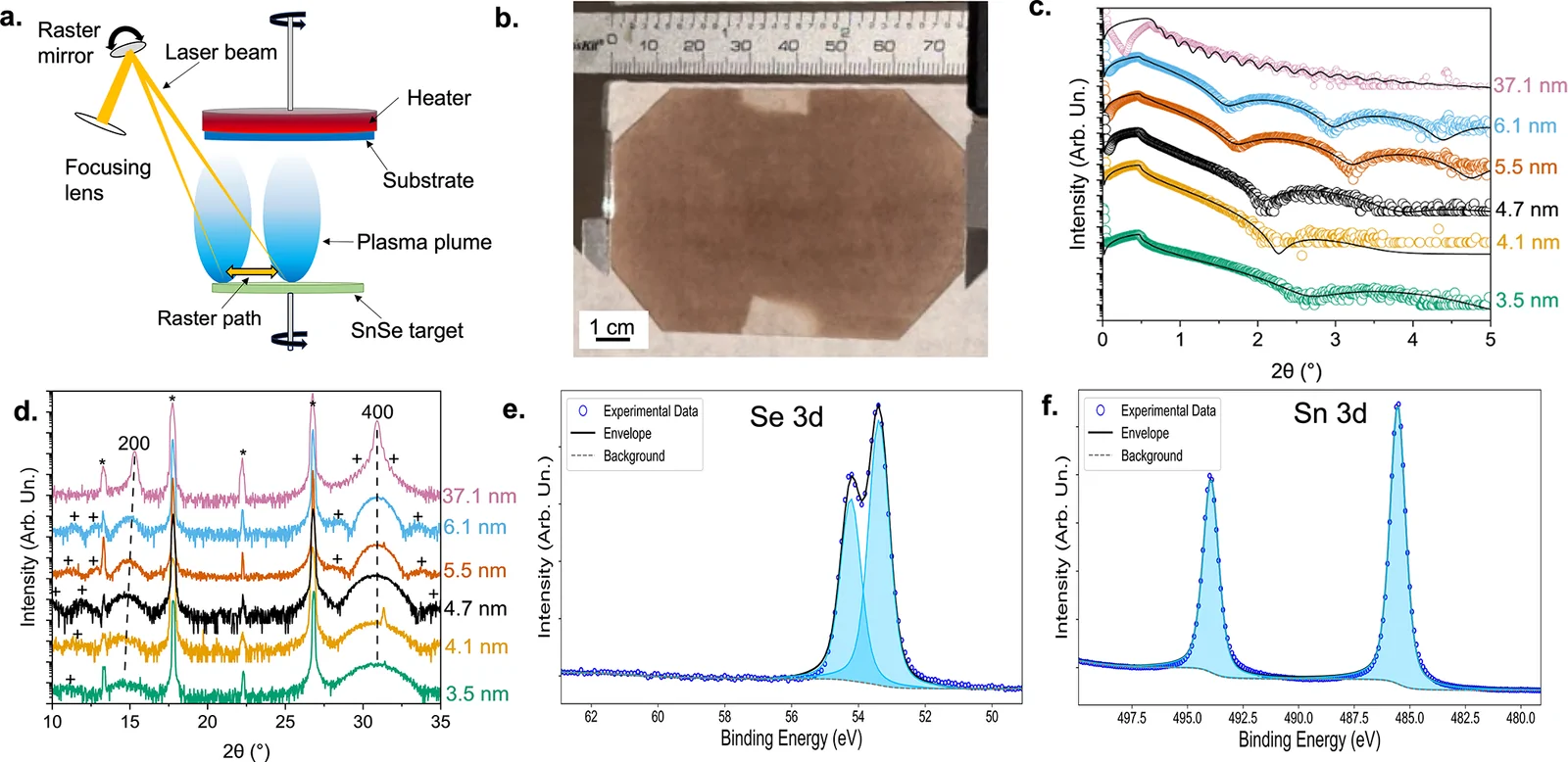
Thickness-controllable deposition of SnSe by mirror-rastered pulsed-laser deposition. (a) Schematic of how mirror rastering enables wafer scale coverage by PLD. (b) Image of a SnSe thin film deposited by mirror rastering across a 7.5 × 5 cm wafer. (c) X-ray reflectivity (open circles) and fits (black line) used to determine the film thickness. (d) Thickness-dependent X-ray diffraction of SnSe showing the consistent phase and orientation control. *2h00* peaks correspond to *Pnma* SnSe, * corresponds to substrate peaks, and + indicates Laue oscillations. (e) XPS of the Se 3d and (f) XPS of the Sn 3d spectral ranges after a cluster argon ion etch.

Once the thickness tunability is established, it is critical to assess how the crystallinity is preserved as thickness is reduced. Figure 1d shows the crystallinity for the films measured by X-ray diffraction (XRD). Across all the film thicknesses measured, the XRD is consistent with (200) oriented *Pnma* SnSe. No other secondary phases or orientations are observed. Furthermore, Laue oscillations (labeled with a +) are observed around the *2h00* SnSe peaks. The presence of Laue oscillations indicates consistent thickness and composition uniformity with smooth film–substrate interfaces.^33^ The XRD also shows a thickness dependent shift in the d-spacing of the 200 and 400 peaks. This thickness-dependent shift in the XRD data is consistent with prior observations of thickness-dependent changes in the van der Waals spacing and in-plane bond lengths in SnSe.^9^ The crystalline quality is further quantified by the rocking curve analysis of the 400 SnSe peaks, as shown in Figure S4. A narrow rocking curve fwhm below 0.15° is maintained from 37.1 to 4.1 nm, demonstrating the film mosaicity remains minimal even as thickness is reduced below 10 monolayers. As the thickness is reduced to 3.5 nm, the rocking curve fwhm increases modestly to 0.21° as interface effects become more pronounced. Notably, the rocking curve fwhm for all samples is the lowest for SnSe films thinner than 20 nm and similar to films of 50–100 nm,^23,24^ underscoring the crystalline quality achieved by PLD. A 3.5 nm film thickness does not represent a limit for the thickness of SnSe. For example, Figure S5 shows the XRD, XRR, and AFM characterization of SnSe films with 2.6 nm thickness which still exhibits (100) oriented *Pnma* SnSe down to 4–5 monolayers. However, at or below these thicknesses, lateral discontinuities begin to form as seen in the AFM micrographs in Figure S5c. Reducing the deposition rate could provide a route to reduce void formation in thinner films to approach the monolayer limit by PLD.^34^ Overall, the XRD results confirm that the crystallinity of the SnSe is maintained near the monolayer limit.

In addition to maintaining the crystal structure, it is also critical to ensure that a 1:1 Sn:Se stoichiometry is maintained from the PLD target. The composition of the films was measured by using X-ray photoelectron spectroscopy (XPS). A self-limiting and stable surface oxide is present in all films prior to etching, which is consistent with previous reports on self-arresting tin oxide formation on SnSe when exposed to the atmosphere.^35^ This oxide has a thickness dependence, attributed to differences in surface roughness, grain morphology, and differences in inter- and intralayer bond strength with reduced dimensionality, as described in a previous study.^35^ Figure 1e,f shows the XPS data and fits for Se 3d and Sn 3d for etched SnSe thin films, respectively. The ratio of Sn:Se is approximately 48:52, which is within the XPS quantification uncertainty of the expected 1:1 ratio.

While XRD shows the average crystallinity across a 1 cm lateral size, it is important to verify that crystallinity is maintained across the entire wafer scale. The phase and film quality across the wafer were thus verified using Raman spectroscopy. Figure 2a shows the Raman spectra taken on a 4.5 nm thick SnSe film at 0.75 cm intervals across the substrate. All Raman spectra are consistent with the *Pnma* phase of SnSe. At the edge of the substrate, a SnSe_2_ secondary phase appears, which is attributed to nonuniform heating at the edge of the substrate. The edge temperature effects could be resolved by using a larger heating chuck. The A_g_ and B_3g_ peaks are assigned according to those in refs 19 and 36 . No other phases are observed by Raman spectroscopy. Referenced to the expected wavenumber positions for bulk *Pnma* SnSe,^19^ ,^36^ A_g_^4^ is blue-shifted by 5 cm^–1^, while B_3g_^1^ and A_g_^3^ are red-shifted 4 and 3.5 cm^–1^, respectively. The Raman shifts of the films with respect to bulk SnSe are consistent with previous reports of the thickness-tunable bonding of SnSe.^21,36^ The Raman peaks of the films shift with respect to single-crystal SnSe, as shown in Figure S6. The shift in peak positions between the film and the single-crystal reference is indicated for each peak. The peak shifts are consistent with previous reports of the thickness-tunable bonding of SnSe.^21,36^ The red shifts of A_g_^3^ and B_3g_^1^ modes correspond to the expansion of in-plane bonds, while the A_g_^2^ red shift corresponds to an expanded van der Waals separation. The A_g_^4^ blue shift indicates contracted out-of-plane covalent bonding.^9^ Thus, having a consistent Raman peak position and structure is indicative of a consistent film thickness. Since the Raman response of these SnSe films does not vary, in either peak position or relative intensity across the wafer, the films must be consistent in phase and in thickness across all measured areas. The consistent film quality is clear from the limited change in the peak position, broadness, or variation in the relative peak ratios across the substrate.

**Figure 2:**
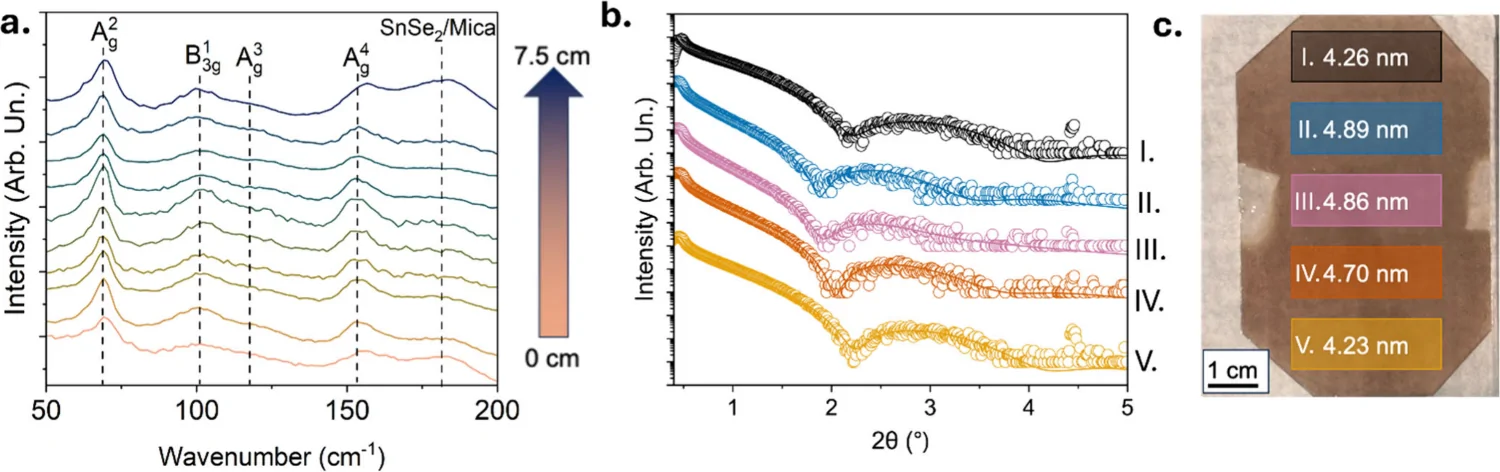
Demonstrating uniform coverage of SnSe across a 7.5 × 5 cm area. (a) Raman spectra of SnSe across the long edge of the wafer. (b) X-ray reflectivity data (open circles) and fits (lines) of SnSe films deposited with the mirror raster PLD process across the substrate. (c) Image of the sample presented in Figure 1b overlaid with the XRR determined thicknesses from (b).

To confirm and quantify the consistent thickness across the length of the wafer, five equally spaced XRR measurements were measured across the wafer. Figure 2b gives the XRR data and fit across the wafer. The lateral size of the X-ray beam was 1 cm, and the position of the measurements across the wafer is displayed in Figure 2c. The sample thickness closely matches the expected thickness from the number of pulses with the average thickness of the wafer being 4.59 nm (∼8 ML) and a standard deviation of 0.29 nm.

To evaluate the impact of raster range on uniformity, films deposited with a reduced raster range were compared in Figure S7. In these samples, the thickness gradient increases from ∼1 ML up to ∼ 3ML when the raster range is decreased by half. This comparison highlights the critical role of mirror rastering in achieving wafer-scale thickness uniformity. Increasing the raster range increases the plume density toward the edge of the substrate, therefore reducing the thickness gradient across the substrate length. Additional increases in the lateral scale and uniformity of SnSe films are anticipated by increasing the raster range. Furthermore, the increased uniformity with increasing raster range suggests that this approach can be expanded to larger wafers. Together, these results confirm that phase and thickness are consistent across the wafer scale.

To further characterize the continuous coverage and uniform morphology of the PLD films, an AFM image across a 0.5 × 0.5 μm and 10 × 10 μm area is presented in Figure 3a,b, respectively. No exposed substrate regions are observed within the scanned area, and no grain boundaries are observed. The AFM shows that the film is continuous and near single-layer surface step height across the measured area. Three distinct step heights are observed across the image. The step height between the layer heights ranges from 0.50 to 0.62 nm, which matches with the expected step height between SnSe monolayers (0.575 nm).^9^ Figure S8b presents line profiles extracted from the AFM scan seen in Figure 3a (Figure S8a). Height distributions constructed from the AFM height data in Figure S8c show that the area of the film is dominated by the mean thickness of the film at 95% with less than 3% and 2% for the two other layer heights observed across the image. The *R*_q_ of the film is 0.29 nm across 100 μm^2^, less than a monolayer of SnSe (0.575 nm). This level of surface roughness is approximately an atomic layer thick, approaching the measured roughness of the bare mica substrate. The low surface roughness, film morphology, and sharp interfaces indicated by the Laue oscillations suggest layer-by-layer growth of SnSe. Reducing deposition rate or postdeposition annealing could provide a route to reduce void formation in thinner films to approach the monolayer limit by PLD.^34^

**Figure 3:**
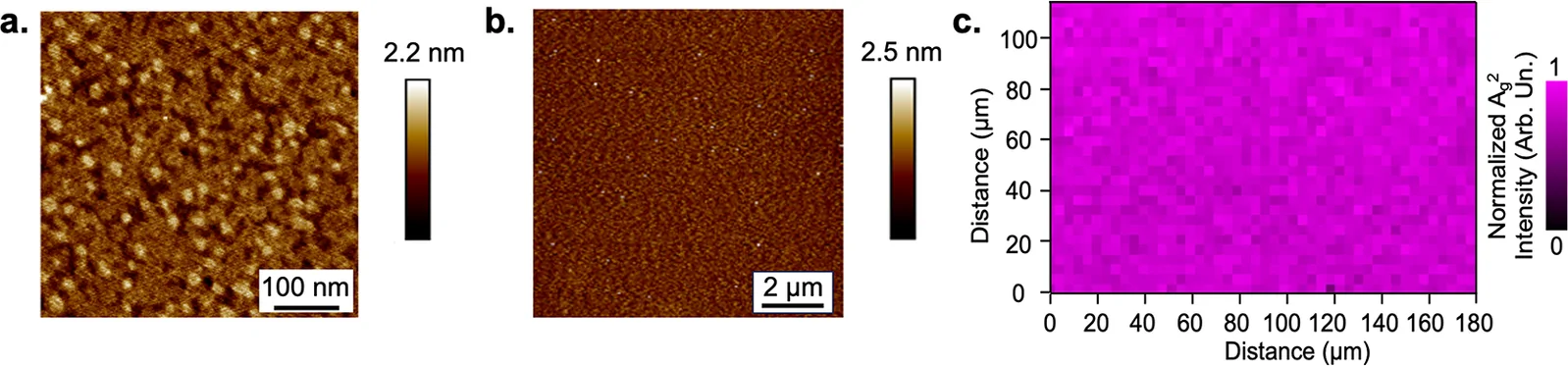
Continuous film thickness of SnSe. (a) Atomic force micrograph of a 6.1 nm thick SnSe film across a 0.5 × 0.5 μm scan area. (b) Atomic force micrograph of a 6.1 nm thick SnSe film across a 10 × 10 μm scan area. (c) Representative Raman map across a 180 × 110 μm area marking the intensity of the A_g_^2^ Raman mode of SnSe.

Figure 3c provides further insight into the minimal spatial variation across the sample. Raman maps are taken over a 180 × 110 μm area with an ∼2 μm spot size with the normalized A_g_^2^ peak intensity mapped in Figure 3c. *A*_g_^2^ is associated with an out-of-plane vibrational mode and is consistent with the wavenumber reported in the literature.^36,37^ Across this measured area, all regions show *Pnma* SnSe. Taken along with the complementary AFM measurements, we show that the SnSe films deposited by mirror-raster PLD do not form in the island morphology previously observed in SnSe films deposited by physical and chemical vapor deposition.^21,38–41^ No voids were observed, indicating continuous coverage of *Pnma* SnSe. Again, these Raman results are indicative of consistent thickness, phase, and film quality across the mapped region. Together, AFM, XRR, Raman, and XRD provide confirmation that the thickness is consistent at the wafer-scale and can be modulated with near-monolayer precision while maintaining low surface roughness and high crystallinity.

In addition to mapping the thickness and structural uniformity across the wafer, it is also critical to ensure that the material is functionally consistent. Here, sheet resistance mapping provides a demonstration of the consistency in the resistance across the wafer. Figure 4a provides a map of the sheet resistance taken across a 25 nm thick SnSe thin film on mica. The sheet resistance holds constant with an average of 0.52 GΩ/sq across a 6 cm span with a standard deviation of 0.13 GΩ/sq. At the edges of the substrate, the sheet resistance deviates from the center with an increase in measured sheet resistance. The difference in electrical properties is understandable given the slight deviations in crystallinity and thickness at the substrate edges (Figure 2a). The edge effects observed here could be resolved with a larger heater and target used for deposition. The resistance measurements demonstrate that not only is the thickness consistent but also the electrical performance is consistent at the wafer scale. Figure S9 shows sheet resistance as a function of SnSe film thickness for samples on mica. As film thickness increases from 4.1 to 8.1 nm, the resistance decreases markedly from 37.3 to 0.2 GΩ/sq. The increased resistance of the SnSe films is attributed to two primary reasons: (1) the increase in band gap with decreasing film thickness,^9^ which reduces intrinsic carrier concentration, and (2) increased surface scattering in thinner films. According to the Fuchs Sondheimer model, a main reason for the generalized increase in electrical resistance as thickness decreases is the increased collision of conduction electrons with the top and bottom boundaries of the film.^42,43^ Additionally, thickness scaling has been shown to modify in-plane bonding and van der Waals spacings,^9^ which may further impact electrical anisotropy. The complex electrical properties of 6 monolayer thick SnSe were probed via electrical impedance spectroscopy. The impedance of SnSe was fit using an equivalent circuit shown in Figure S10, with contact resistance, and a constant phase element (CPE) and resistor in series (Table S1). A CPE was used due to the depression of the Nyquist plot.^44^ The near-unity value of the CPE exponent is indicative of a uniform device response. The Debye-type relaxation of the impedance is consistent with what has been observed for isostructural SnS.^45^

**Figure 4:**
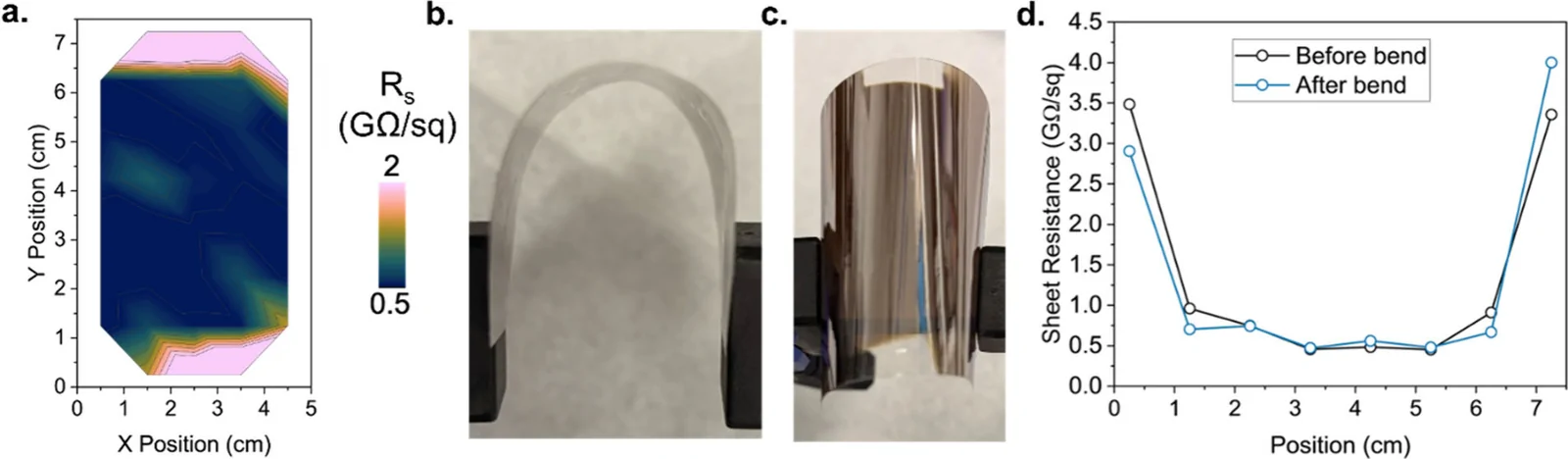
(a) Sheet resistance as a function of position of 25 nm thick SnSe film on a mica substrate. (b, c) Images of bending an SnSe film on a muscovite mica substrate with a bend radius of 1.3 cm. (d) Sheet resistance of the SnSe film before and after bending.

An additional benefit of using a mica substrate is flexibility, as devices on mica substrates have shown stability with a bending radius below 1 cm and >10^3^ cycles.^46,47^ Figure 4b,c shows the flexibility of the SnSe thin films on mica, demonstrating the potential for flexible electronics. The bend radius of the substrate pictured is 1.3 cm. The sheet resistance measured before and after bending shows consistent resistance (Figure 4d) with a measured average resistance of the inner 6 cm being 0.64 GΩ/sq, within the standard deviation measured before bending.

In conclusion, laser mirror rastering pulsed laser deposition enables wafer-scale coverage of SnSe with monolayer thickness control and atomic scale surface roughness. XRD, XRR, and AFM show that the SnSe structure and thickness are consistent across the mica wafers. Raman spectroscopy shows peaks matching the *Pnma* phase of SnSe films that do not vary in peak position or relative intensity across the wafer, further indicating consistent phase and thickness across all measured areas. The monolayer-precision control demonstrated here enables the deliberate selection of even- or odd-layer-number films, which is a prerequisite to accessing the noncentrosymmetric properties predicted for few-layer monochalcogenides. Sheet resistance mapping shows that the electrical response matches the consistency of the structure on the wafer scale. Furthermore, the preserved electrical performance after mechanical flexing shows that mirror-raster PLD is a viable route to integrate 2D monochalcogenides into flexible nanoelectronics. Overall, these results demonstrate a processing method for the integration of highly anisotropic 2D chalcogenides into field effect transistors, optoelectronics, and photonics.

## Supplementary Material



## Notes

Additional information, including the pulsed laser deposition conditions, X-ray diffraction measurement specifications, X-ray reflectivity measurement and fitting parameters, Raman spectroscopy measurement specifications, X-ray photoelectron spectroscopy with cluster etching details, four point probe sheet resistance measurement details, in plane Mica/SnSe XRD figure showing in plane growth orientations, XRD scans as a function of temperature and laser fluence, atomic force microscopy scans of samples deposited at 475 and 500 °C, thickness dependent rocking curves, XRD, XRR, and AFM scans for a 2.6 nm SnSe film, a figure showing the spectral shifts of a 4.6 nm SnSe on mica scan with respect to a bulk SnSe single crystal, a schematic showing initial mirror raster thickness variations, additional AFM analysis of step heights from the scan in Figure 3a, a graph showing sheet resistance as a function of SnSe film thickness, and electrical impedance spectra and fits for a 4.1 nm thick SnSe thin film (PDF)
