# Supplementary material for: Wafer-Scale Deposition of 2D SnSe with Monolayer-Thickness Precision
Source: Nano Lett. 2026 Aug 20;26(35):11664–70. doi: 10.1021/acs.nanolett.6c01711 (PMC13564916; doi:10.1021/acs.nanolett.6c01711)
Supplement: Supplementary file 1 [file nl6c01711_si_001.pdf]

Supporting Information

Wafer-Scale Deposition of 2D SnSe with Monolayer-Thickness Precision

Lauren M. Garten,[1*] Hannah E. Wolfe,[1] Jeremy Knight,[1] Jonathan R. Chin,[1] Joshua D. Wahl,[1] Marshall B. Frye[1]

[1]School of Materials Science and Engineering, Georgia Institute of Technology, Atlanta, Georgia 30332-0245, USA

Methods

Deposition

Thin films of SnSe are deposited using pulsed laser deposition (PLD). A PVD Products pulsed laser deposition system equipped with a KrF (248 nm) excimer laser (Coherent Compex 205F) with a mirror raster optics train. The base pressure of the system prior to deposition was held below 5 x 10^{-7} Torr. The fluence of the laser at the target is 1.6 J/cm^2, corresponding to an input laser energy of 203 mJ. Samples were deposited on cleaved 2.5 x 2.5 cm and 7.5 x 5 cm muscovite mica substrates (MTI Corp., hi-grade). Substrates were rinsed with isopropanol after cleaving and subsequently adhered to the substrate holder using silver paste (Ted Pella Inc., Fast-Drying Silver Paint) to ensure thermal conductivity between the heater and substrate. Silver paint was subsequently cured at 350 °C in atmosphere on a hot plate. Deposition temperature ranged from 450 °C - 475 °C. The temperature was measured with a thermocouple on the heating chuck, therefore the substrate temperature is likely lower. Samples were held at deposition temperature for 5 minutes prior to deposition. Samples were cooled to 25 °C before removing from the deposition chamber to avoid oxidation.

Thin film characterization

X-ray diffraction (XRD) was measured with a Rigaku Smartlab XE system with a copper $K_{\alpha 1}$ source (1.54 Å) and an incident beam Ge (220) two bounce monochromator. The system is also equipped with a high-resolution θ–θ closed-loop goniometer. The 2θ scans were measured with a scan speed of 4 °/min and a step size of 0.02°/step. The same measurement setup was used to measure the X-ray reflectivity (XRR) of the samples to determine thickness, density, and roughness of the samples, with a 1 °/min scan rate and 0.02 °/step. The thickness was determined by fitting the X-ray reflectivity data using the Rigaku Smartlab Studio II in adherence to the following equation:

$$Sin^2\theta_i = \theta_c{}^2 + \frac{(n_i + \Delta n)^2\lambda^2}{4t^2} \qquad \text{Eq. 1}$$

where t is the thickness, λ is the X-Ray wavelength (Cu $K_{\alpha 1}$ source = 1.54 Å), n_i is the index of the Kiessig fringe maxima, θ_i is the angle of the Kiessig fringe maxima, and θ_c is the critical angle.[1] XRR was used for the thickness measurements in this study because it has previously been shown to have high precision and accuracy.[1,2]

Raman spectroscopy was measured with a Invia Qontor Renishaw Raman with a 785 nm laser, 2400 lines/mm grating, silicon CCD detector, and a lateral resolution <1 µm. The spectral shifts are referenced to scans from a SnSe bulk single crystal, purchased from HQ Graphene. Atomic force microscopy (AFM) was measured with a Bruker Icon AFM. Scanasyst air tips (Bruker, Si tip on nitride lever, k = 0.4 N/m, tip radius = 2 nm) were used for all scans. A scan speed of 0.5 Hz and 512 samples/line were used for imaging the samples. Samples were imaged in quantitative nanomechanical mapping mode to differentiate between the substrate and film.

The composition of the films was measured using X-ray photoelectron spectroscopy (XPS). A Thermo K-Alpha XPS with a monochromated Al K_α (1.486 keV) source and 180° double-focusing hemispherical analyzer are used for the measurements in the study. The SnSe films were etched using a cluster source (Thermo, Magcis), with an energy of 8,000 eV and a cluster size of 2,000 atm/cluster for 90 seconds. A flood gun was used to reduce the effects of charging and surface XPS scans were charge-shift corrected to the adventitious C 1s peak at 284.80 eV.[3] The thickness of the sample presented in Figure 1e and 1f of the manuscript is 28.1 nm.

Four-point resistance measurements are taken on a 25 nm thick SnSe film using a Keithley 2450 source meter unit and a Signatone s-302-4 at intervals of 0.9 cm in the x and y directions. Sheet resistance using a procedure from [4]. Figure S7 shows sheet resistance as a function of SnSe film thickness. The electrical impedance spectra were measured with a Modulab XM MTS system (Solartron) with a femto-ammeter module and a frequency range of 1 MHz to 1 Hz. The electrical impedance spectra was then fit using ZView 4 using the equivalent circuit shown in Figure SXa. The X^2 value was held below 0.0003 for the fits to ensure goodness of fit.

Supplementary Figures

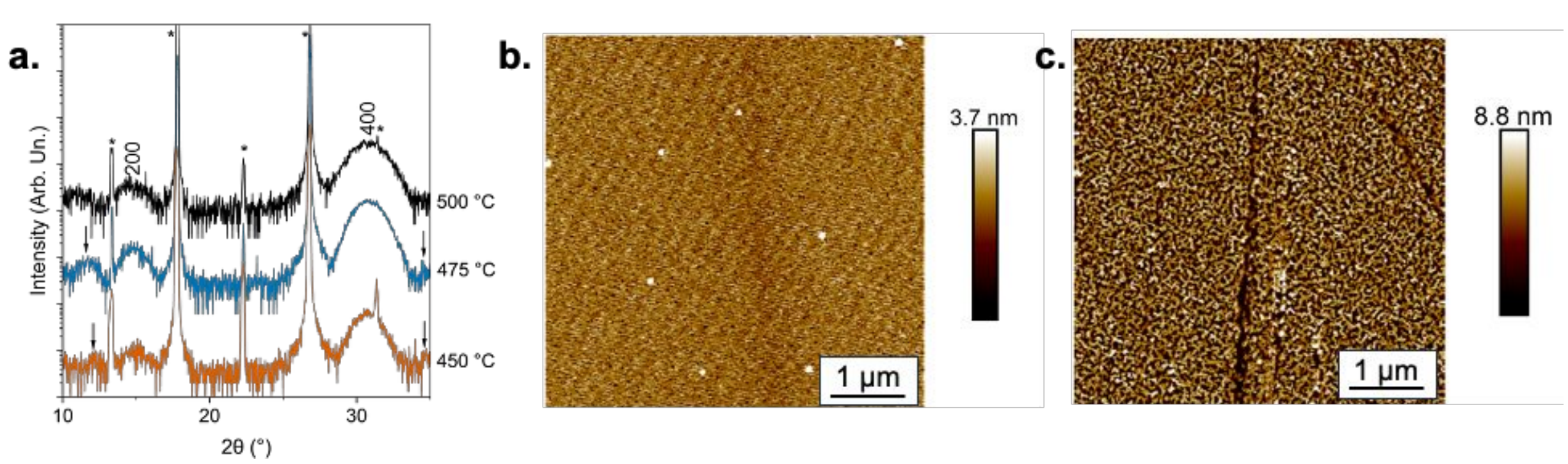

Figure S1. a. X-ray diffraction of SnSe deposited by PLD as a function of deposition temperature. *2h*00 peaks are attributed to *Pnma* SnSe, * peaks are from the muscovite mica, and arrows indicate Laue oscillations. Atomic force micrographs of SnSe films deposited at **b.** 475 °C and **c.** 500 °C.

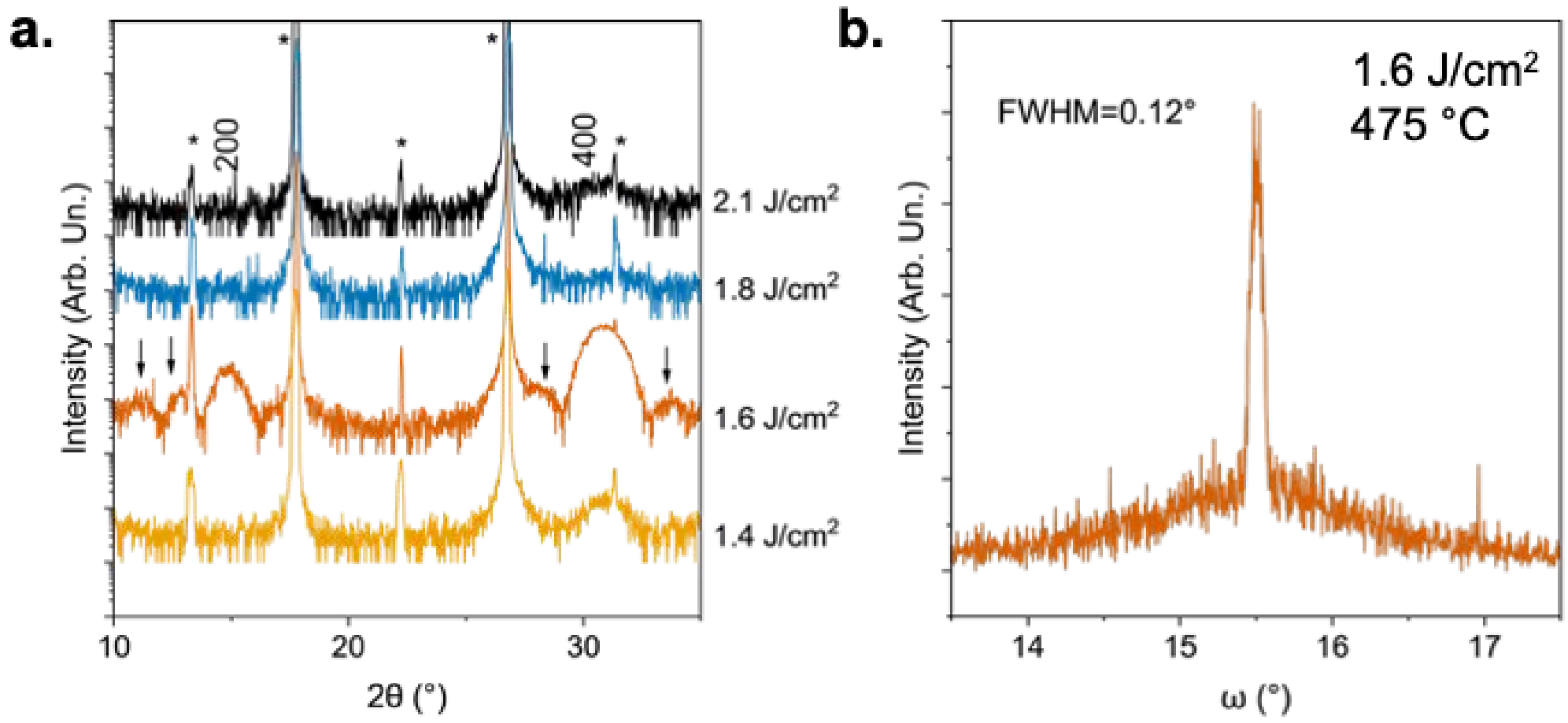

Figure S2. **a.** X-ray diffraction of SnSe films as a function of laser fluence. *2h*00 peaks are attributed to *Pnma* SnSe, * peaks are from the muscovite mica, and arrows indicate Laue oscillations. **b.** Rocking curve of an SnSe film deposited at the optimized temperature and fluence (1.6 J/cm^2, 475 °C).

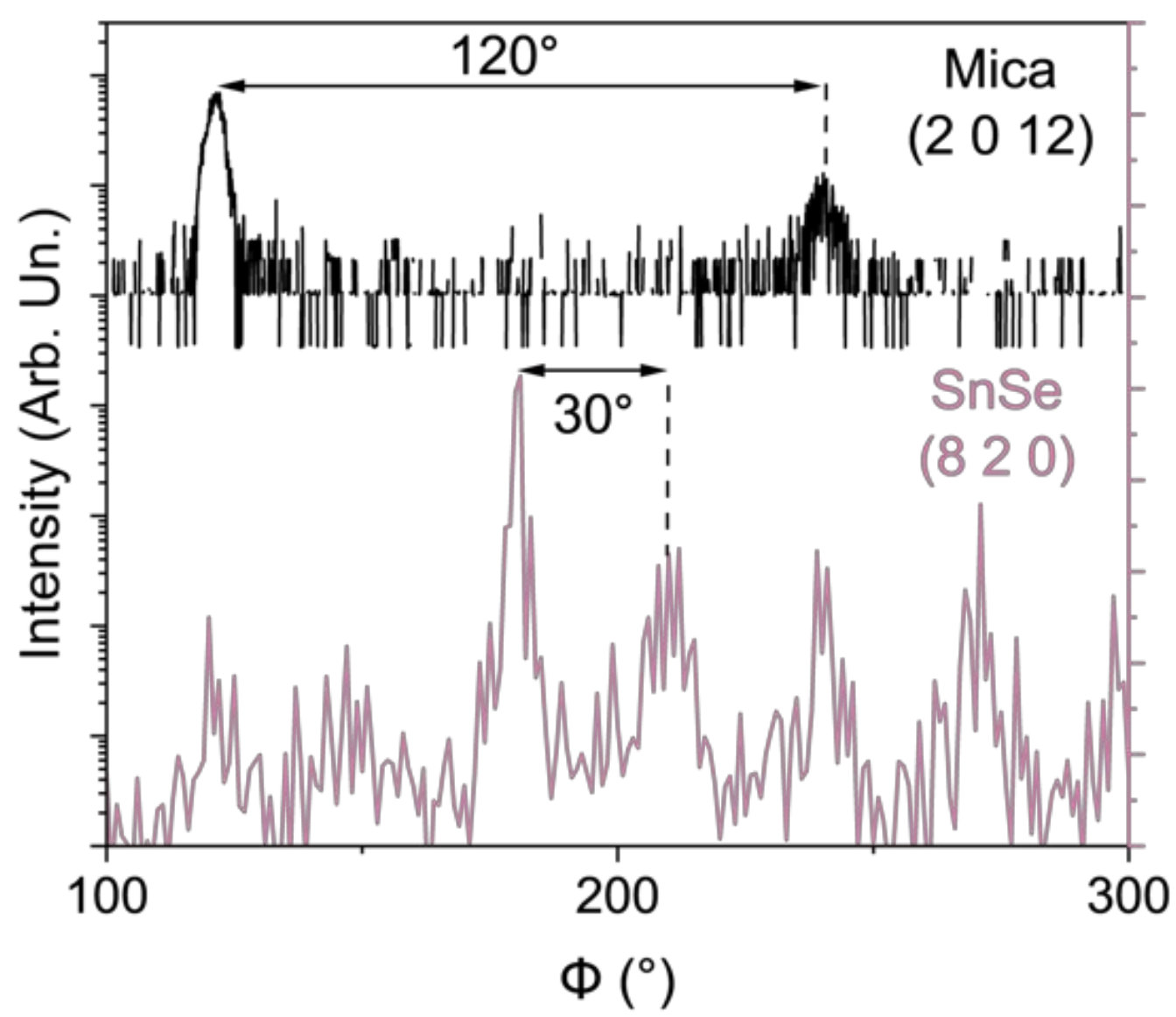

Figure S3. The in-plane XRD of Mica (top) and SnSe (bottom). There are 6 distinct in-plane growth orientations of SnSe that are aligned with the underlying substrate

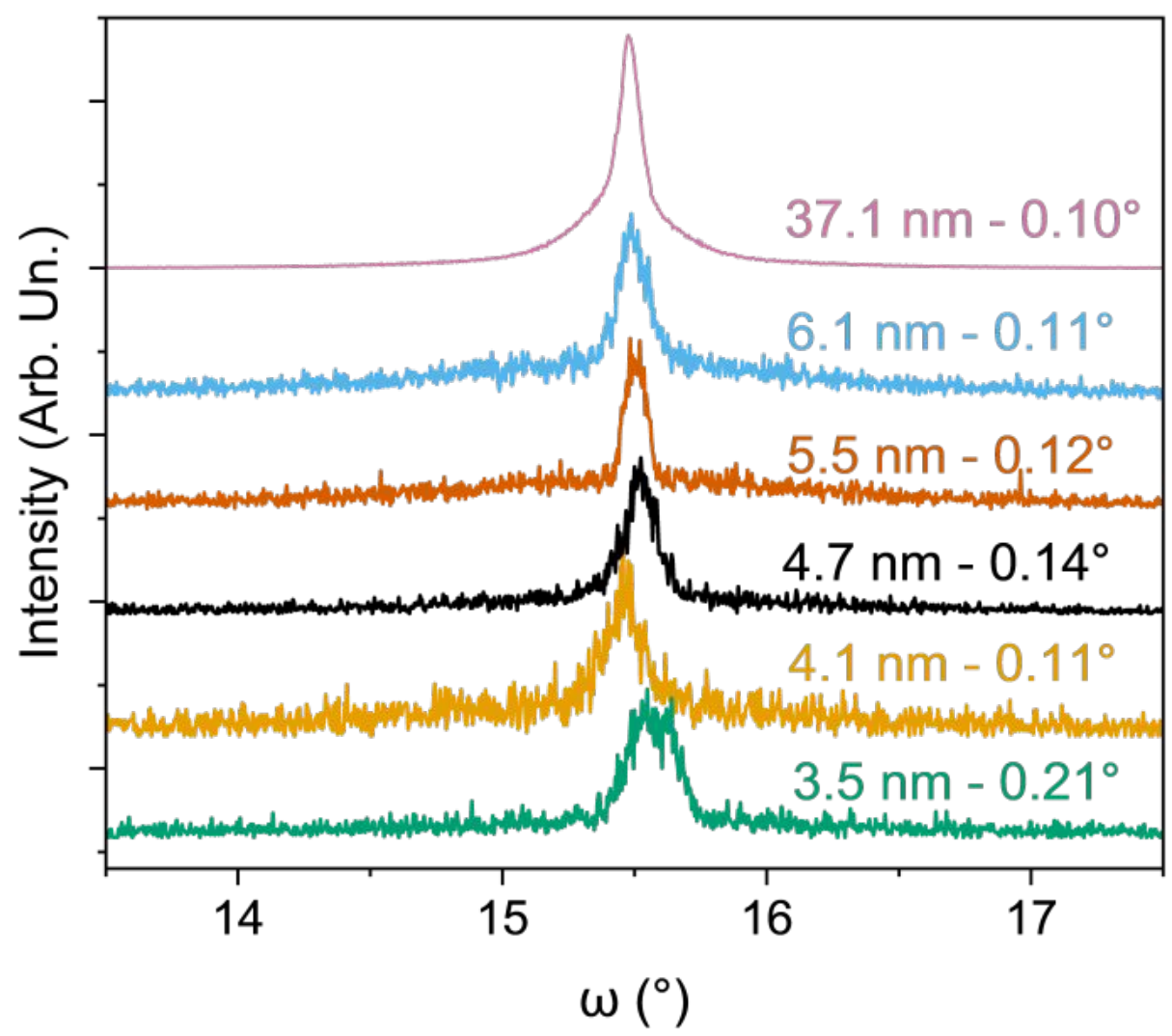

Figure S4. Thickness-dependent rocking curve measurements of the SnSe 400 peak. The full-width at half-maximum of each thickness is marked on the plot.

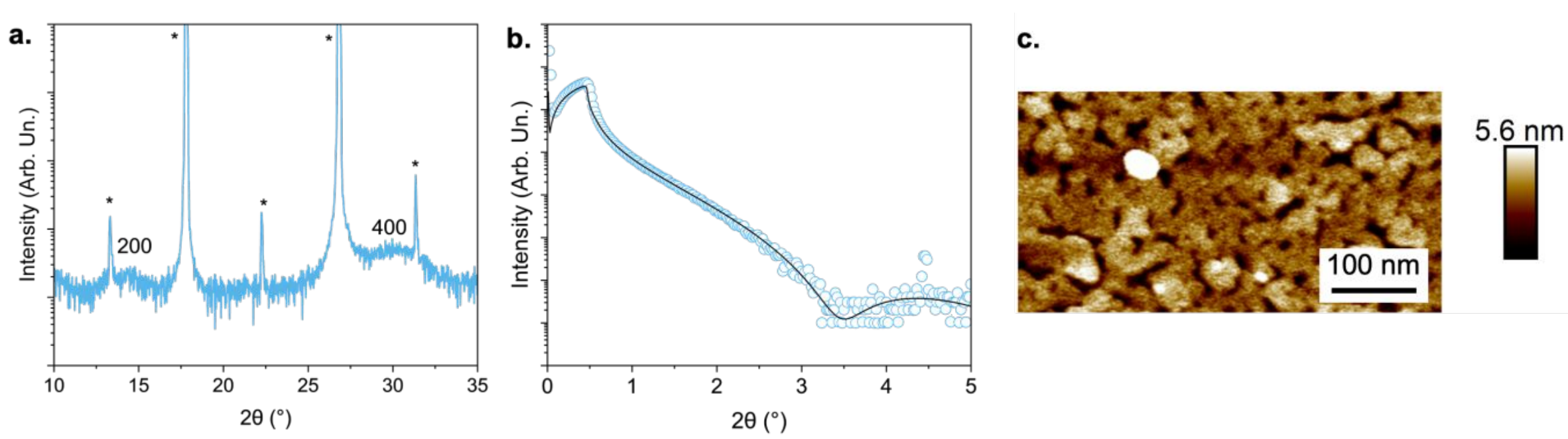

Figure S5. a. X-ray diffraction for 2.6 nm SnSe film, **b.** X-ray reflectivity for a 2.6 nm SnSe film, and **c.** Atomic force microscopy of a 2.6 nm thick SnSe film.

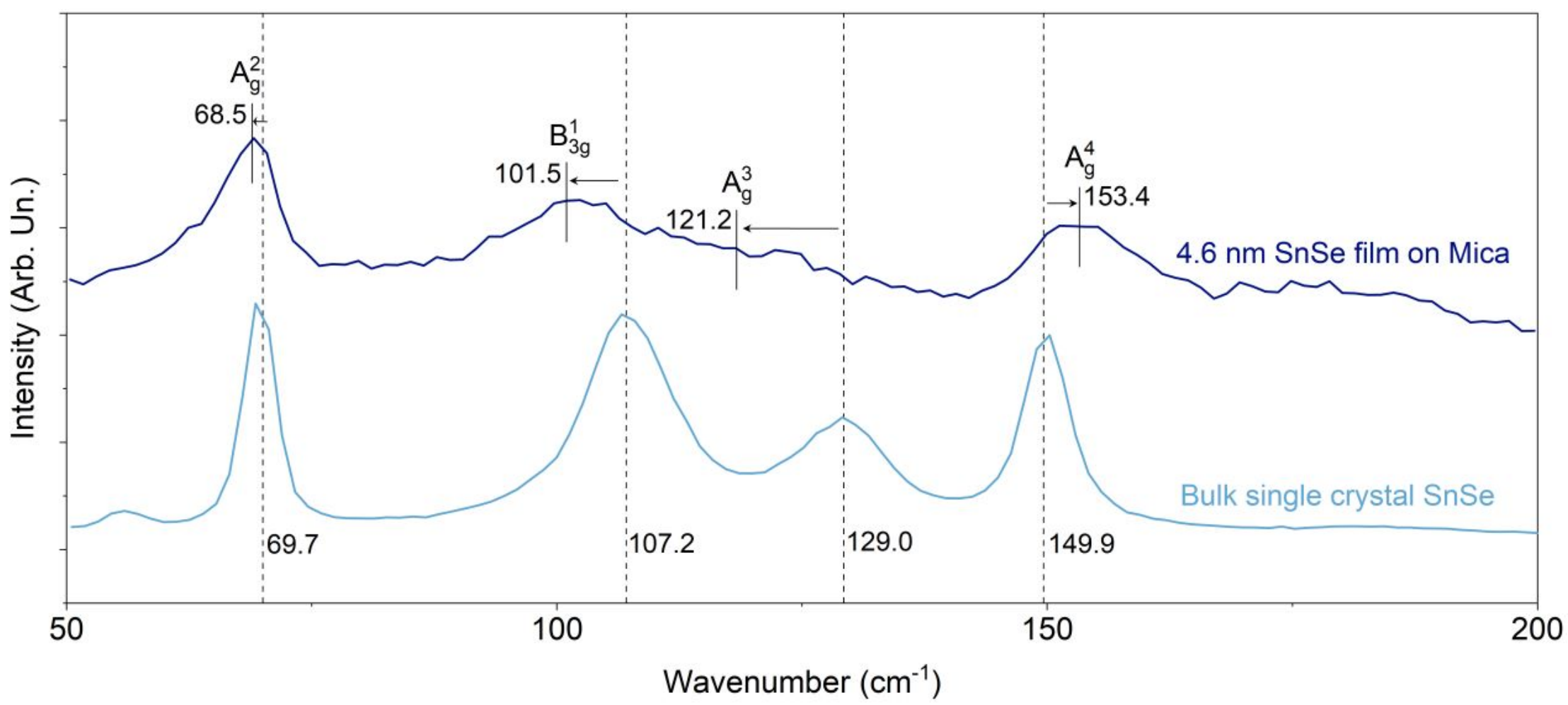

Figure S6. Raman scan of a 4.6 film overlayed with Raman scan of a SnSe bulk single crystal. The arrows indicate the shift in peak position of the thin films with respect to the bulk SnSe single crystal.

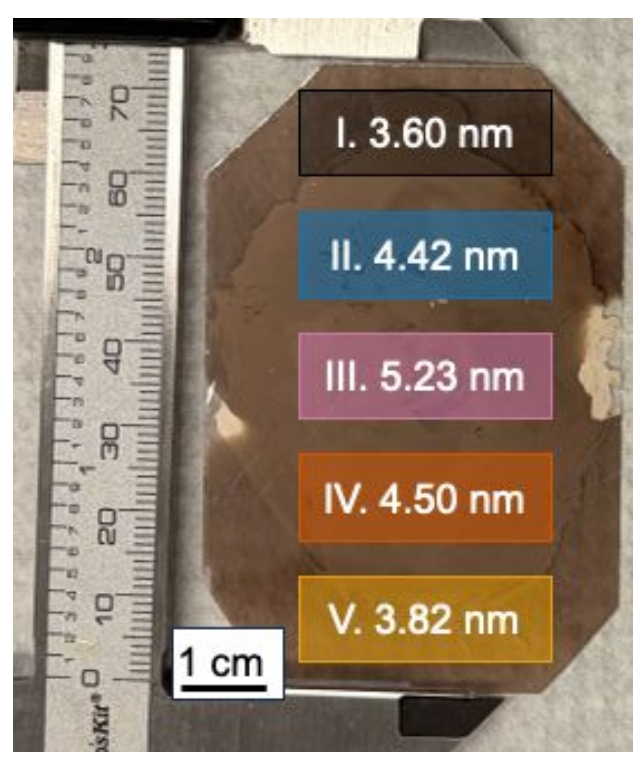

Figure S7. Thickness variation across an SnSe film 7.5 x 5 cm wafer using the initial mirror-raster conditions.

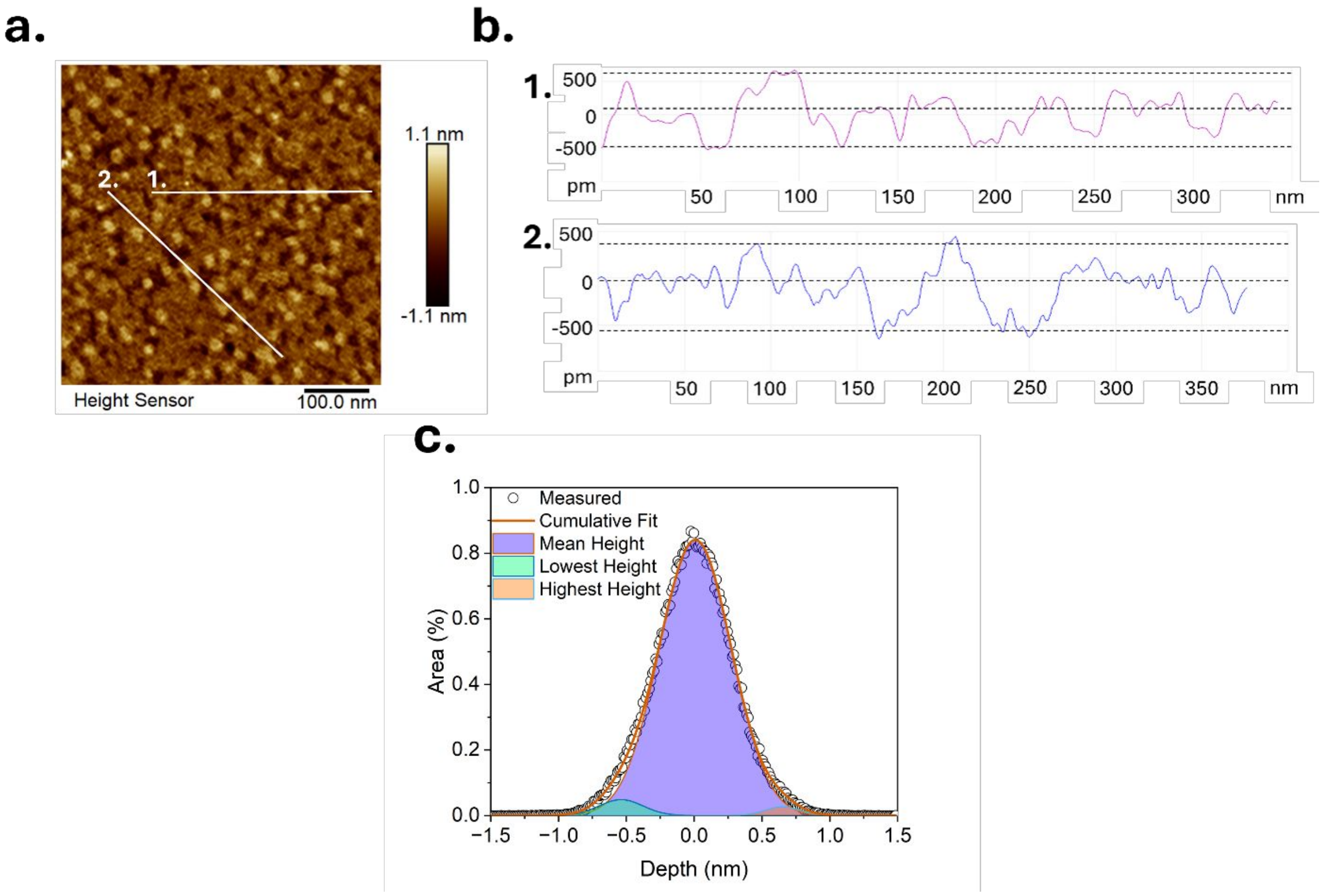

Figure S8. a. AFM micrograph (also shown in Figure 3a) with lines indicating where the line scan height profiles were taken. **b**. Height profiles across the lines shown, with dashed lines indicating the bounds for a monolayer thickness. **c**. Height distribution across the measured sample, showing that the height of the 6.1 nm thick sample has a thickness range <2 nm, with a distribution of three different layer heights.

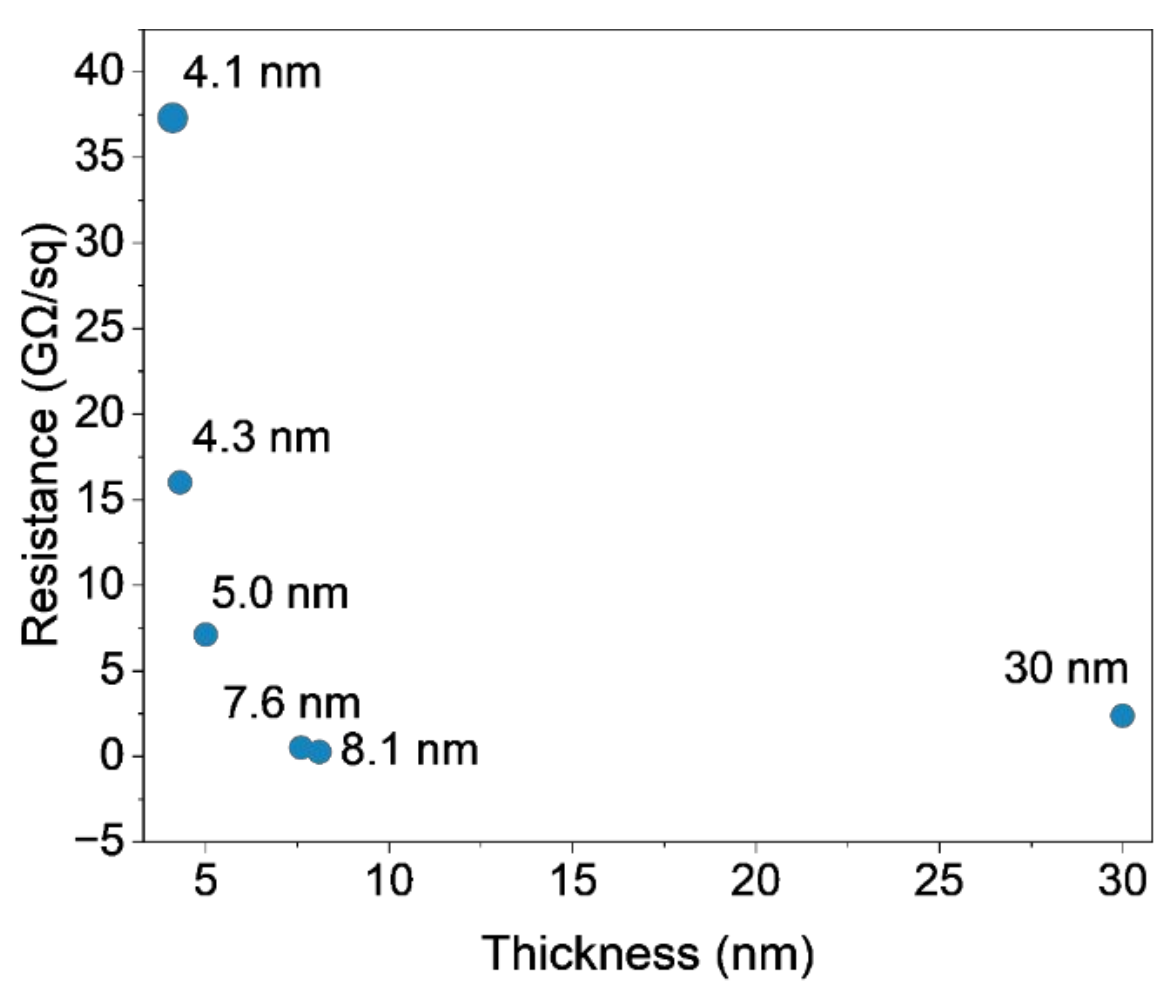

Figure S9: Sheet resistance as a function of film thickness for SnSe films on mica.

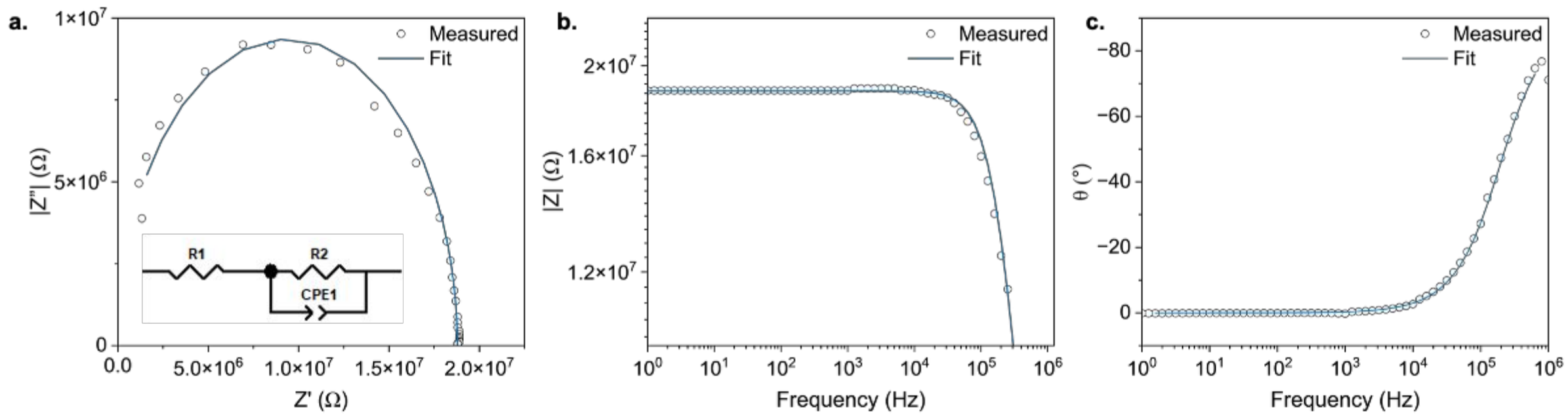

Figure S10. Measured electrical impedance spectra and associated fits of a 4.1 nm thick SnSe film. **a.** Nyquist plot; **b.** Bode magnitude plot; and **c.** Bode phase plot.

Table S1. Extracted circuit elements from the electrical impedance spectra of a 4.1 nm thick SnSe film.

Label	Meaning	Values
R1	Contact resistance	0.004 Ω
R2	Film resistance	1.88×10^{7} Ω
CPE 1	SnSe constant phase element	T = 4.56×10^{-14} Ω
		P = 0.99

References

1. Miller, A. M. *et al.* Extracting information from X-ray diffraction patterns containing Laue oscillations. *Z. Für Naturforschung B* **77**, 313–322 (2022).

2. Huang, T. C., Gilles, R. & Will, G. Thin-film thickness and density determination from x-ray reflectivity data using a conventional power diffractometer. *Thin Solid Films* **230**, 99–101 (1993).

3. Swift, P. Adventitious carbon—the panacea for energy referencing? *Surf. Interface Anal.* **4**, 47–51 (1982).

4. Smits, F. M. Measurement of Sheet Resistivities with the Four-Point Probe. *Bell Syst. Tech. J.* **37**, 711–718 (1958).
